# A comparison of mandibular and maxillary alveolar osteogenesis over six weeks: a radiological examination

**DOI:** 10.1186/1746-160X-10-50

**Published:** 2014-11-28

**Authors:** Marthinus J Kotze, Kurt-W Bütow, Steve A Olorunju, Harry F Kotze

**Affiliations:** Department Maxillo-Facial and Oral Surgery, Faculty of Health Sciences, University of Pretoria, Pretoria, South Africa; College of Health Sciences, University of KwaZulu-Natal, Durban, South Africa; Medical Research Council of South Africa, Pretoria, South Africa; Faculty of Health Sciences, University of The Free State, Bloemfontein, South Africa

**Keywords:** Regeneration, Alveolar bone, Radiological, Gray-scale

## Abstract

**Introduction:**

Insufficient information exists on comparing radiological differences in bone density of the regeneration rate in the alveolar bone of the maxilla and mandible following the creation of similar defects in both.

**Methods:**

Alveolar bone defects were created from five healthy Chacma baboons. Standardized x-ray images were acquired over time and the densities of the selected defect areas were measured pre-operatively, directly post-operatively and at three- and six weeks post-operatively. Differences in densities were statistically tested using ANOVA.

**Results:**

The maxilla was significantly more radiologically dense (p = 0.026) than the mandible pre- operatively. No differences were obtained between the maxilla and mandible directly postoperatively and three- and six weeks post-operatively respectively; i.e. densities were not significantly different at the different time points after the defects had been created (three weeks: t = 1.08, p = 0.30; six weeks: t = 1.35, p = 0.19; three to six weeks: t = 1.20, p =0.25). The increase in density in the mandible was 106% (8.9 ± 7.6%/time versus 4.3 ± 2.7%/time) over three weeks, 28% (15.0 ± 8.1%/time versus 11.7 ± 8.0%/time) over six weeks and 56% (12.5 ± 9.7%/time versus 8.0 ± 6.9%/time) over three-to-six weeks and was higher than in the maxilla over the same intervals.

**Conclusions:**

Radiological examination with its standardized gray-scale analysis can be used to determine the difference in bone density of the maxilla and mandible. Although not statistically significant, the mandible healed at a faster rate than the maxilla, especially observed during the first three weeks after the defects were created.

## Introduction

Surgical procedures in the alveolar processes of the maxilla and mandible often lead to permanent loss of bone substance. This missing bone has to regenerate to restore normal function. Bone is distinctive in connective tissue healing because it heals entirely by cellular regeneration and production of a mineral matrix. The sequence of events that occurs after tooth extraction has been described previously in detail [1]. At an extraction site, the alveolar socket is filled with a blood clot, which is replaced by vascularized granulation tissue. During this regeneration process, intense osteoclastic bone resorption occurs to remove necrotic bone and bone debris. At the same time, osteoblast differentiation and proliferation start. New bony trabeculae are formed in the apical region at day five. Bone apposition in the extraction socket occurs along the lateral alveolar walls and at the fundus of the socket by means of new woven bone projecting until the socket is entirely filled between days 20 and 28. Bone remodeling occurs finally when the newly formed woven bone is replaced by mature lamellar bone [2]. No literature could be found on a comparison of the regeneration rate of the mandible and maxilla following trauma. In one study, the overall healing process following tooth extraction in the mandible and maxilla was evaluated but the rates of regeneration were not compared [2]. Different aspects of alveolar bone remodeling were investigated. Remodeling of alveolar bone subjected to orthodontic forces was evaluated and reported in the literature [3–6]. In one study significantly more orthodontic tooth movement was observed for maxillary than for mandibular teeth for the same time of force application. In the same study an overall decrease in bone volume for the first four weeks following the application of the orthodontic force and an increase in bone formation rate after 12 weeks were found [3]. Guided bone regeneration was also investigated in procedures such as bone augmentation [7, 8], through the use of different membranes [9, 10], and in implantology through observing osseointegration as well as bone quality [11–13]. Furthermore, investigations of alveolar regeneration in the mandible and maxilla were done using resorbable and non-resorbable plates and screws [14, 15].

When alveolar cortical bone density of the maxilla was compared with that of the mandible [16], the mandibular measurements were statistically significantly higher than those of the maxilla, except at the incisor region measurements. For cancellous bone, the canine and retromolar areas of the mandible were statistically higher than those of the maxilla [16]. Huja *et al*. found that the average bone volume within the alveolar process of the mandible was 2.8 fold greater than in the maxilla [4].

The process of bone regeneration can be monitored radiologically as bone density becomes more detectable during the regeneration process [1, 17, 18]. A significant correlation between mineral bone density and bone structure exists when density is measured radiologically [19]. This correlation enables the rate of healing for the maxilla and mandible to be determined. A previous histomorphometric study indicated that the rate of healing of the mandible was approximately twice as fast as that of the maxilla [4]. However, there are no radiology studies that could substantiate these findings. Owing to the lack of information in comparing the healing rate after trauma between the maxilla and mandible, a radiological evaluation in determining alveolar bone density differences between the mandible and maxilla was decided on as an assessment tool.

## Methods

Five healthy male Chacma baboons (*Papio ursinus*) were used. The average weight of the animals was 19.8 ± 4.3 Kg, which implied it is young animals, as the weight of an adult male Chacma baboon can reach up to 40 Kg in five years [20]. Approval for the study was granted by both the Animal Use and Care Committee (AUCC), and a subcommittee of the Committee for Research Ethics and Integrity at University of Pretoria and North West University. The animals were anaesthetized with intravenous ketamine hydrochloride (dose: 10 mg/kg). In order to control haemostases and pain, 1.8 mL (9 mg) Bupivacaine with 0.5% epinephrine 1:200,000 (as bitartrate) (Novocol, Pharmaceuticals of Canada. Inc., Cambridge, Ontario, Canada) was injected intramuscularly.

### Surgical intervention

Defects were created in the premolar areas of the mandible and maxilla by the same surgeon. The bone at the selected site being exposed by reflection of the overlying mucosa. Three alveolar bone defects, 3 mm deep, were created with a 3 mm diameter trephine bur, fitted onto a straight surgical hand piece connected to a surgical drilling unit. The defects were positioned 2 mm apart.

### Radiology

**Figure 1:**
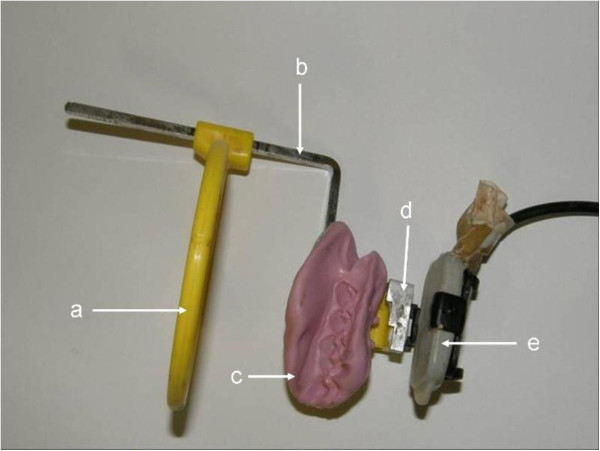
**The apparatus after a lab putty impression was made.** This impression was made to ensure that images were acquired in the same position every time an image of that quadrant was made. (a + b = Rinn apparatus; c = #21 mandible disposable impression tray with Lab Putty impression; d = aluminum step wedge; e = Gendex Visualix EHD Digital intra-Oral x-ray unit – Size 1 censor (universal size) attached to a XPC-DS Digital Position System.

Radiographs were taken pre-operatively, directly post-operatively and again after three and six weeks. Standardized reproducible radiographs for analysis was acquired for each of the four quadrants at each time point with an apparatus (Figure 1) constructed as follows: For each quadrant, maxillary and mandibular in each animal, a sectional tray was prepared by cutting in half a disposable mandibular impression tray (#21, Wright Cottrell Co., Kingsway West, West Dundee, Dundee). In each instance the tray was adjusted to fit properly over as many teeth as possible. A bite block ( XPC-DS Digital Position System (Gendex, Lake Zurich, Illinois) was secured onto the tray with a self-tapping screw and cyanoacrylate cement. The block carried the cradle for the sensor (Gendex Visualix EHD Digital intra-Oral x-ray unit – Size 1 (universal size) with 25.6 line pairs/mm; KaVoDental, Gendex Imaging, Via Alessandro Manzoni, 44, 20095 Cusano Milanino, Milan, Italy) (Figure 1). A step wedge was made from a 3 mm x 6 mm strip of commercially available aluminium. Three steps were cut, 2 mm wide and 1 mm deep. The wedge was supported on the bite block so that it was parallel to and just touching the sensor. Removal and accurate replacement of the wedge in this position was achieved by an arrangement of location pins which fitted precisely into receptacle holes. A drill of the requisite diameter was used to make two small holes through the aluminium and into the bite block. Short straight sections of a paperclip were cut and glued into the holes in the bite block. These protruding pins fitted precisely into the holes in the aluminium wedge, enabling repeated removal of the wedge and its subsequent replacement in the same position. The procedure was repeated for every bite block, enabling ready transfer of the aluminium wedge for each radiograph. The bite block, sensor and tray were secured to a Dentsply-Rinn apparatus (Dentsply, Elgin, Illinois), consisting of a metal ring holder and plastic positioning ring.

The sectional tray was loaded with laboratory putty (Coltene/Whaledent, Switzerland), a silicone base and polysiloxane activator and positioned in the quadrant to include as much of the alveolar ridge as possible. While the laboratory putty was still in the soft stage of the setting process, an x-ray was taken to enable confirmation that the sensor was correctly positioned. Once the putty had set, the impression of the teeth and the alveolar ridge provided a secure key to accurate repositioning of the set up for subsequent radiographs. The position of the ring on the holder as well as the position in the bite block was identical for all the radiographs.

A Planmeca Intra Wallmount X-ray unit (Planmeca Oy, Asentajankatu 6, 00880, Helsinki, Finland.) was used to acquire the radiographs which were taken at 8 mA with 63 kV and an exposure time of 0.08 seconds. A Toshiba D-0711 SB x-ray tube was used and the focal spot was 0.7 × 0.7 mm. The focal distance for all the images was 110 mm.

The computer software program used for the radiology was Gendex VixWin Pro (Gendex Dental Systems 901 West Oakton Street Des Plaines, IL 60018).

### Evaluation methods

**Figure 2:**
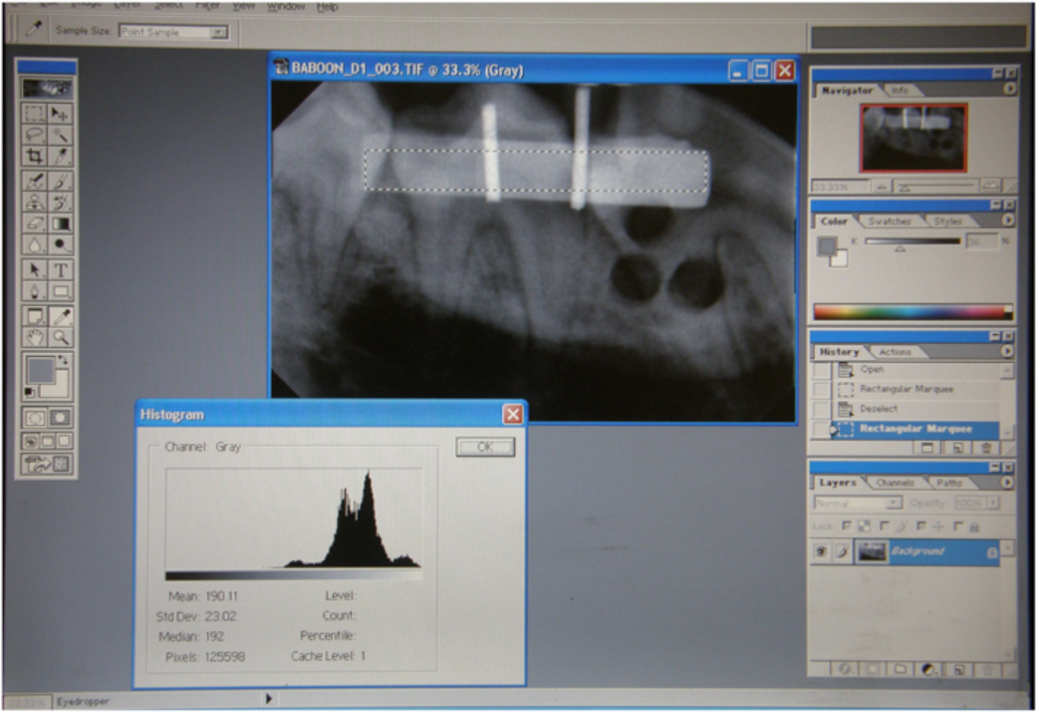
**A histogram of a selected area on the aluminum step wedge.**

**Figure 3:**
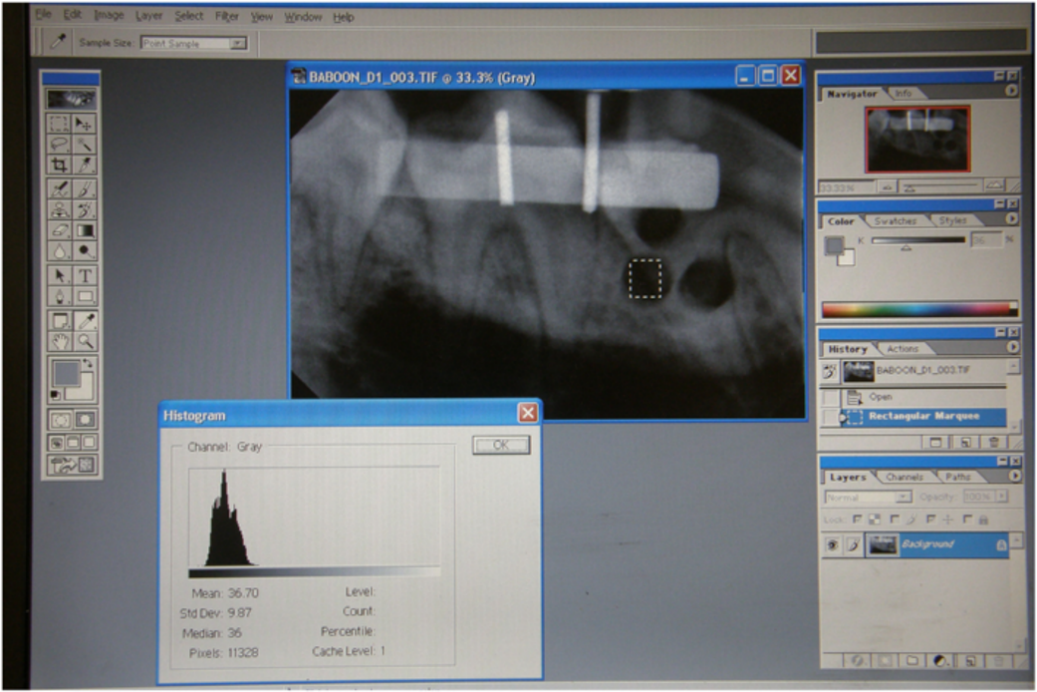
**A histogram of a selected area on the created defect.**

Four digital images were acquired per quadrant on each occasion when records were taken i.e. 16 images per animal at each of the four time periods. The images were imported into Adobe Photoshop (V6.0; Adobe Systems Inc., San Jose, CA). An A4 transparent sheet was positioned on the computer screen and firmly secured. The first pre-operative image was imported on the screen and with the lasso tool of the program an area of interest (AOI) was selected on the image of the step wedge (Figure 2). The corners of the selected area were marked on the transparent sheet with a fine point permanent marker pen. Now each of the three postoperative images could sequentially be accurately positioned and oriented on the screen, using the drag and drop function, so that the marks on the transparent sheet precisely superimposed on the selected area on the image. The gray scale values for this defined area of the wedge were standardized across all four images recorded from each quadrant by using the histogram, contrast and brightness tools of the Photoshop software [21] to make the required point adjustments, ensuring that the density never differed by more than 12 data points Hence all images now reflected comparable degrees of gray scale. An AOI was selected on each of the biopsy sites on each of the images taken immediately post-operatively and marked on the transparent sheet (Figure 3). The average gray-scale values for these areas on each of the three defects on each image were determined and recorded.

This was done for all images of a quadrant acquired from each of the different time periods. The method used was described in a 2012 publication of Kotze *et al*. [17].

### Statistical analyses

All the animals were within the same age, weight range and sex. Only one examiner took the measurement and recorded the results. The analysis of the data was standardized and repeatable which implies that there was no reason to measure inter-observer variation. ANOVA for repeated measures (analysis of variance considering more than one factor period) was used to test for possible differences in the data set. The variance ratio (F) can be defined as ratio of the effect of treatment to the unexplained variance is assumed to have F distribution. Periodic changes (actual and percentage) were analyzed using the two-sample t-test within each time point to compare mandible and maxilla. The t-statistic is a ratio of the departure of an estimated parameter (mean difference between Mandible and Maxilla in each time period) its standard error and is defined as the ratio of the difference between changes in the measurements at two time intervals and the associated standard error.

## Results

The results are summarized in Table 1. The calculations of the percentage change at three and at six weeks included the pre-operative values to exclude the effect of differences in bone density of the different animals. Through this approach, the pre-values were equal to zero and the percentage change expressed as the increase from pre-values.

**Table 1:** **The percentage changes in grey-scale values over six weeks**

	Grey-scale values		Per Cent Change over Time		
	Pre-operative	Post-operative ^a^	Three weeks ^b^	Six weeks ^c^	Three to Six weeks ^d^
Maxilla	98 ± 26	54 ± 19	4.3 ± 2.7	11.7 ± 8.0	8.0 ± 6.9
Mandible	72 ± 22	53 ± 18	8.9 ± 7.6	15.0 ± 8.1	12.5 ± 9.7

^a^Post-operative change = (preoperative - postoperative) ÷ preoperative x 100.

^b^Three weeks = (3 weeks - postoperative) ÷ preoperative x 100.

^c^Six weeks = (6 weeks - postoperative) ÷ preoperative x 100.

^d^Three/six weeks = (3/6 weeks - postoperative) ÷ preoperative x 100.

Values are given as a mean ± 1SD.

With the use of ANOVA for repeated measures, there was a significant difference between the densities in the maxilla and mandible (F = 11.92, p = 0.0007) when the data were analyzed. No significant differences between animals were found (F = 2.18, p = 0.24). Periodic analysis indicated that the maxilla was significantly denser than the mandible before the defects were created (t = 2.4743, p = 0.0235). No significant difference was found between the post-operative densities of the maxilla and mandible (t = 1.3417, p = 0.20). Analysis showed that the percentage change in densities was not significantly different at any of the time points after the defects were created (three weeks: t = 1.08, p = 0.30; six weeks: t = 1.35, p = 0.19; three-to-six weeks: t = 1.20, p = 0.25). The rate of increase in density in the mandible was 106% (8.9% over three weeks versus 4.3% over three weeks), 28% (15.0% over six weeks versus 11.7% over six weeks) and 56% (12.5% over three to six weeks versus 8.0% over three-to-six weeks) more for the maxilla after three, six and three-to-six weeks respectively.

## Discussion

Bone regeneration in the premolar area of the maxilla and mandible using changes in average gray-scale value (AGV) derived from radiographs was investigated. The radiological changes in density of bone were compared with histological and histomorphometrical measurements and a correlation were found between the different approaches [22–24].

This principle of change in radiological density over time can be used as an indicator of bone turnover. The pre-operative AGV indicated that the maxilla was significantly denser than the mandible (Table 1; F = 11.92, p = 0.0007). This finding contrasts with previous findings that the density of the mandible is higher than that of the maxilla [25]. This can be due to the difference in position of the AOI, as the bone density measured on radiographs in the mouth is normally affected by surrounding structures in the length of the pathway of the x-ray and also the thickness of bone [22]. The AOI in our study was more apically located and included more of the dense palatal bone on the radiographic image. However, in the present study, the pre-operative difference in bone density of the maxilla and mandible is not of any significance, as the changes in density were measured over time with the post-operative value as the starting point. This pre-molar area included more bone volume and the denser soft tissue from the palate, as well as the thicker cortical bone of the palate. The pre-operative values were used for calculating the percentage change in bone density. The possible influence of differences in bone density between the maxilla and mandible and between animals before the defects were created was excluded by calculating the changes relative to the pre-operative densities. Through this approach, the post-operative values were normalized to zero. It was not surprising that the post-operative percentage density for the area of interest was similar for both the mandible and maxilla following the removal of the same volume of bone (Table 1). Any change to a number greater than the post-operative percentage AGV was regarded as a percentage increase in bone regeneration.

The increase in density of the mandible after the surgical intervention – was not significantly different from that in the maxilla at three-, six- and three-to-six weeks (Table 1). This finding contrasts with that of a study using histomorphometric methods where the mandible healed twice as fast as the maxilla [26]. Similar results were found in our study comparing the rate of healing between the mandible and maxilla during the first three weeks period. The percentage increase in density in the mandible was 8.9% and 4.3% in the maxilla (Table 1) which represents a difference of approximately 106%. At six weeks the difference in percentage of the rate of healing between the mandible and maxilla decreased to an average of 20%. The time sequence of bone regeneration following an extraction is started with clot formation on the same day which is replaced by granulation tissue in the first week. The granulation tissue is replaced by connective tissue on day 20. Osteoid formation is evident from day seven and on day 38, two-thirds of the extraction socket is filled by connective tissue [27]. The sequence of the regeneration process indicates active cellular activity directly post-operative in the defect area which implies a quicker increase in density of the AOI on the radiological image. The tempo of the process of regeneration decreases from day 20 as connective tissue and osteoid is developing. As the mandibular alveolar bone is denser than the maxillary alveolar bone, it can be expected that more regeneration tissue will be present in the mandible bone, therefore the difference in the rate of healing between the maxilla and mandible in the first three weeks. The mandible is subjected to higher mechanical forces and consequently has a higher rate of healing than the maxilla [27]. The dynamism imposed by muscle force on the bone causes complex patterns of stress and strain in the mandible, such as sagittal and transverse bending and deformation from shear and torsion [28]. In contrast, the maxillary and pre-maxillary bones are primarily exposed to forces generated by occlusal contact with the mandibular teeth [29]. The bone quality for both anterior and posterior jaw regions are predominantly types 2 and 3 (Lekholm-Zarp classification) [30]. The anterior part of the mandible has the densest bone, followed by the posterior mandible, anterior maxilla, and posterior maxilla [31]. The area of interest was the pre-molar region of both the mandible and maxilla. Both regions were Lekholm-Zarp type II or III classification with the density of the cortical and trabecular bone in the mandible the highest [13, 16]. One may speculate that the denser quality of bone in the mandible provided more bone cells for osteogenesis at the site of trauma. Bone cells respond to mechanical stimulation and induce new bone formation *in vivo* and also increase the metabolic activity and gene expression of osteoblasts [31]. However, the molecular events involved in the translation of mechanical stimulation into cell proliferation and bone formation are not yet well understood [32]. The more mechanical stimulation of the mandibular bone cells due to the denser bone and more forces influencing the mandible [28, 29] may also contribute to the increase in the rate of bone healing, especially following the creation of defects at the postoperative three-week period.

## Conclusions

There was no statistically significant difference in the density of the alveolar bone between the maxilla and mandible. Therefore, bone regeneration in the mandible and maxilla after similar defects were inflicted to both and a radiological evaluation carried out three weeks and six weeks post-operatively was not different. In contrast, the rate of regeneration of the mandible after three weeks was 106% higher than that of the maxilla. The literature review provided no studies that compared the rate of bone regeneration following the inflicting of defects without the need of osteointegration or stimulation by foreign biomaterials. With the aid of histology or histomorphmetry as a control, further investigation with a larger sample over longer periods of time is necessary before a definite conclusion can be reached on a comparison of alveolar bone regeneration of the mandible and maxilla.
